# How can we better use Twitter to find a person who got lost due to dementia?

**DOI:** 10.1038/s41746-018-0017-5

**Published:** 2018-04-18

**Authors:** Kelvin K. F. Tsoi, Nicholas B. Chan, Felix C. H. Chan, Lingling Zhang, Annisa C. H. Lee, Helen M. L. Meng

**Affiliations:** 1 Stanley Ho Big Data Decision Analytics Research Centre, The Chinese University of Hong Kong, Sha Tin, Hong Kong; 2 Jockey Club School of Public Health and Primary Care, The Chinese University of Hong Kong, Sha Tin, Hong Kong; 3 School of Journalism and Communication, The Chinese University of Hong Kong, Sha Tin, Hong Kong; 4 Department of Systems Engineering and Engineering Management, The Chinese University of Hong Kong, Sha Tin, Hong Kong

**Keywords:** Communication, Public health

## Abstract

Twitter is a social media platform for online message sharing. The aim of this study is to evaluate the effectiveness of using Twitter to search for people who got lost due to dementia. The online messages on Twitter, i.e., tweets, were collected through an Application Programming Interface. Contents of the tweets were analysed. The personal characteristics, features of tweets and types of Twitter users were collected to investigate their associations with whether a person can be found within a month. Logistic regression was used to identify the features that were useful in finding the missing people. Results showed that the young age of the persons with dementia who got lost, having tweets posted by police departments, and having tweets with photos can increase the chance of being found. Social media is reshaping the human communication pathway, which may lead to future needs on a new patient-care model.

## Social media: Twitter best practices for finding lost people with dementia

Twitter can help find people who get lost due to dementia—if the social media tool is used optimally. Kelvin Tsoi and colleagues from the Chinese University of Hong Kong analysed over 45,000 tweets about people with dementia or Alzheimer’s disease who went missing. The researchers identified 40 individuals who were found within a month of the first tweet, and 14 others who were not. Comparing the two groups, they found that people were more likely to be located if they were younger, if police departments were tweeting about the missing person, and if tweets contained photos of the lost individuals. The findings point toward better ways of using Twitter to track down those who get lost, a common and unpredictable phenomenon that occurs for at least half of all people living with dementia.

## Introduction

Dementia is a syndrome of a complex, irreversible and relentlessly progressive loss of cognitive function.^1^ It was estimated that 35.6 million people lived with dementia worldwide in 2010.^2^ As the global population ages, the number of people with dementia continues to rise^3^ and becomes a public health priority.^4–6^ The most common brain illness that is associated with dementia is Alzheimer’s disease, contributing to 60–70% of all dementia cases.^7^ It is expected 1 in 85 people worldwide will suffer from Alzheimer’s disease by 2050.^8^ Dementia is the major cause of dependence and subsequent care among senior people.^9^ Moreover, there is an increasing awareness of the impact of dementia on people at a younger age.^10^

Getting lost is a common and unpredictable phenomenon for a person with dementia. Previous research indicated the chance of a person who got lost due to dementia or Alzheimer’s disease ranged from 46 to 71%, and they might get lost during usual daily activities.^9,11–14^ In previous studies, the majority of patients with dementia failed to recognise their ways back to the original locations when they get lost due to difficulties in processing route information or even initiating a conversation with other people on the street.^9,11,15^ The consequences of a person with dementia getting lost can thus be hazardous.^1,9,16,17^

Previous studies have shown that traditional news reports could help with finding missing persons with dementia who got lost in the community.^1,18,19^ Therefore, it is believed that social media, such as Twitter, can serve the same purpose. Social media have become one of the largest and fastest information sources worldwide.^20,21^ Twitter is one of the most popular social media platforms with more than 300 million active users per month from a diverse background.^22^ It allows users to post short messages, i.e., tweets, of no longer than 140 characters. In addition, these tweets can include a variety of elements such as external webpage links, photos, videos and keyword search queries, i.e., hashtags. These enormous amount of Twitter data are openly retrievable through the Application Programming Interface (API) provided by Twitter.^23^ Although Facebook is another popular social media platform, only public messages posted on Facebook pages can be retrieved and the connection across user accounts are unavailable.^24^ Therefore, the spread of social media posts cannot be completely traced for a social network analysis.

Here we introduced an epidemiological approach along with the application of data analytics tools to study social media data. A case–control study design in epidemiology was applied to identify the risk factors of whether a person can be found, and retrospectively compared the features of tweets and retweets for each lost person. The impact of using social media was then investigated. As a result, we can suggest better ways of using Twitter to enhance the chance of finding a lost person with dementia.

## Results

### Database

**Fig. 1:**
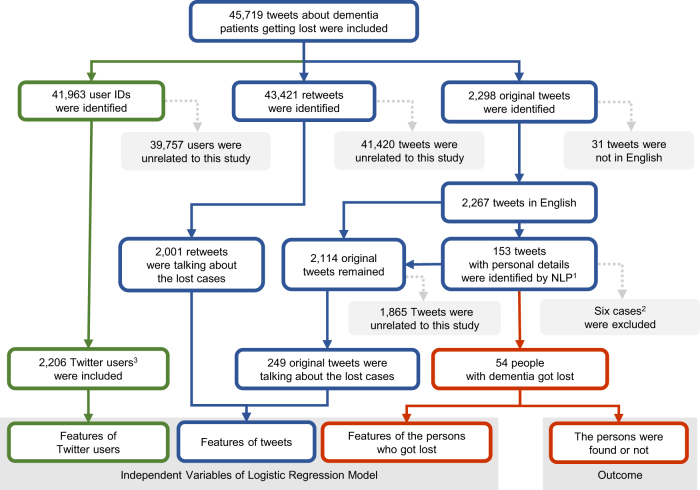
Flow chart of data extraction from Twitter. Boxes with different colours represent different types of data: green for Twitter users; blue for tweets and retweets on Twitter; and red for people who got lost with dementia. ^1^ Natural language processing (NLP) was used to identify name, age and location of the person who got lost from the tweet contents. ^2^ Six people were excluded because one person got lost before the study period, the names of two people could not be identified, and three people were found before the first original tweet. ^3^ Twitter users only included tweet-writers and retweeters. Number of followers were extracted from the individual users

A total of 45,719 tweets about people who got lost due to dementia were collected in April and May 2017 in Twitter (https://twitter.com/). The detailed procedure of data processing is shown in Fig. 1. Among all original tweets, 60 lost people were identified by natural language processing using contents from 153 original tweets. Six of them (10%) were excluded because (i) one person got lost before the study period, but the tweets were posted on early April; (ii) the names of two people could not be identified on Twitter, so their corresponding tweets cannot be recognised or traced; and (iii) three people were found before their first original tweets were posted. As a result, 54 people who got lost due to dementia were included in this study and 40 of them (76.9%) were found within a month of the first original tweet. The 54 people accounted for a total of 249 original tweets from 227 tweet-writers, and 2001 retweets from 1979 retweeters. The average found time (Standard Deviation, (SD)) was 21.4 (30) h, and it ranged from 16.8 min to 140 h.

### Baseline characteristics

Among the 40 people who could be found, i.e., the case group, the mean age was 74.6 years and 32 (80%) of them were male (Table 1). All people were found if they got lost while driving. A total of 199 original tweets and 1828 retweets were disseminated to look for them (Table 2). Among the 183 tweet-writers, 15 of them (8.2%) were from police departments, and 60 tweet-writers (32.8%) were from media organisations. Among the 14 people who cannot be found, i.e., the control group, the mean age was 80.3 years and 8 (64.3%) of them were male. A total of 50 original tweets and 173 retweets were disseminated to look for them (Table 2) within 21.4 h, which was the average found time for people in the case group. Among the 44 tweet-writers, 1 tweet-writer (2.3%) was from police department, and 10 tweet-writers (22.7%) were from media organisations.

**Table 1:** Baseline characteristics of the people who got lost due to dementia

	People with dementia were found	
	Yes	No
**People who got loss due to dementia**		
No. of people who got loss	40	14
Age, mean (SD)	74.6 (6.9)	80.3 (7.5)^a^
Below 70	8 (20.0%)	2 (16.7%)
70–79	23 (57.5%)	2 (16.7%)
80 or above	9 (22.5%)	8 (66.7%)
Male (%)	32 (80.0%)	8 (64.3%)
Country of origin (%)		
United States	32 (80.0%)	10 (71.4%)
United Kingdom	4 (10.0%)	4 (28.6%)
Others	4 (10.0%)^b^	0 (0.0%)
Activity when they got lost (%)		
Driving	10 (25.0%)	0 (0.0%)
Walking	14 (35.0%)	5 (35.7%)
Not available	16 (40.0%)	9 (64.3%)

Remarks:

^a^The ages of two people were not reported in the tweets

^b^The four cases were from Australia, Canada, Malaysia and New Zealand

**Table 2:** Features of Twitter use^a^ among the people who got lost due to dementia

	Total No. (%)			Average no. (SD) per person who got lost		
People with dementia who can be found	Yes	No	*p*-value^b^	Yes	No	*p*-value^c^
Types of Twitter users:						
No. of tweet-writers	183	44		4.6 (5.0)	3.1 (3.6)	0.258
From police departments	15 (8.2%)	1 (2.3%)	0.294	0.4 (0.5)	0.1 (0.3)	**0.009**
From media organisations	60 (32.8%)	10 (22.7%)	0.265	1.5 (1.7)	0.7 (1.1)	0.051
No. of retweeters	1807	172		45.2 (148.8)	12.3 (18.3)	0.178
From police departments	7 (0.4%)	0 (0.0%)	0.884	0.2 (0.5)	0.0 (0.0)	**0.003**
From media organisations	6 (0.3%)	2 (1.2%)	0.312	0.2 (0.4)	0.1 (0.4)	0.950
No. of followers, in thousands	12,338	963		308.4 (749.7)	68.8 (96.4)	**0.031** ^d^
From police departments	388 (3.1%)	9.3 (1.0%)	<**0.001**	3.5 (4.7)	0.7 (2.4)	**0.010**
From media organisations	6314 (51.2%)	474 (49.2%)	0.256	8.9 (4.8)	4.5 (5.5)	**0.013**
Types of Twitter posts:						
No. of original tweets	199	50		5.0 (5.4)	3.6 (3.7)	0.292
From police departments	19 (9.5%)	1 (2.0%)	0.143	0.5 (0.8)	0.1 (0.3)	**0.005**
From media organisations	66 (33.2%)	10 (20.0%)	0.102	1.6 (1.9)	0.7 (1.1)	**0.031**
Tweets with photos	175 (88.0%)	37 (74.0%)	**0.024**	4.4 (5.3)	2.6 (3.5)	0.177
Tweets with website links	190 (95.5%)	44 (88.0%)	0.098	4.8 (5.4)	3.1 (3.7)	0.224
Tweets with hashtags	44 (22.1%)	13 (26.0%)	0.691	1.1 (1.6)	0.9 (1.3)	0.696
Average length of tweets (no. of words)				15.0 (3.8)	17.1 (4.6)	0.136
No. of retweets	1828	173		45.7 (150.1)	12.4 (18.4)	0.176
Retweets from police departments	8 (0.4%)	0 (0.0%)	0.809	0.2 (0.6)	0 (0.0)	**0.044**
Retweets from media organisations	6 (0.3%)	2 (1.2%)	0.308	0.2 (0.4)	0.1 (0.4)	0.950
No. of retweet / each original tweet				10.8 (24.8)	6.1 (9.8)	0.324

Remarks:

^a^All tweets were counted until the time before the people who could be found. The average time to find the people was 21.4 h from the first post of original tweet. This was also used as the cut-off times for the people who could not be found in the control group

^b^*p*-value of *Χ*^2^ test, and significant results (*p*-value <0.05) were shown in bold

^c^*p*-value of independent sample *t*-test, and significant results (*p*-value <0.05) were shown in bold

^d^The number of followers was transformed to logarithmic scale before performing a *t*-test

### Features of tweets

Among the 199 original tweets in the case group, the most commonly used words in the tweets were: missing (197 times), dementia (111 times), alzheimer (82 times), and police (83 times). The average length of tweets (SD) was 15.0 (3.8) words, and the maximum length was 21 words. Similarly, among the 50 original tweets in the control group, the most commonly used words in the tweets were: missing (51 times) and dementia (41 times). The average length of tweets (SD) was 17.1 (4.6) words, and the maximum length was 23 words. Tweets with photos of the persons who got lost were more commonly observed in the case group than that in the control group (88.0% vs. 74.0%, *p*-value of *Χ*^2^ = 0.024), but the tweets with website links or hashtags were comparable between the case and control groups (95.5% vs. 88.0%, *p* = 0.098, and 22.1% vs. 26.0%, *p* = 0.691, respectively) (Table 2). A total of 19 original tweets (9.5%) were posted by police departments in the case group, but only one original tweet (2.0%) was posted in the control group (*p* = 0.143). A total of 66 original tweets (33.2%) were posted by media organisations in the case group, and only ten original tweets were posted in the control group (*p* = 0.102). The average number of tweets posted by police departments in the case group was significantly higher than that in the control group (0.5 vs. 0.1, *p* = 0.005). Similarly, the average number of tweets posted by media organisations in the case group was also significantly higher than that in the control group (1.6 vs. 0.7, *p* = 0.031). The average number of retweet per original tweet (SD) was 10.8 (24.8) in the case group, and 6.1 (9.8) in the control group (*p* = 0.324).

### Factors associated with the person who can be found

**Fig. 2:**
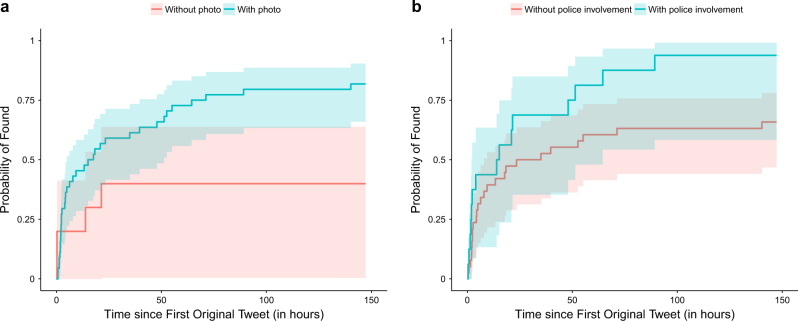
Graphs of Probability of Found against Time since First Original Tweet. The above graphs show the probabilities of found for **a** people with or without any original tweets with photos and **b** people with or without original tweets by police department. Filled region represents the 95% confidence interval of the corresponding curve

**Fig. 3:**
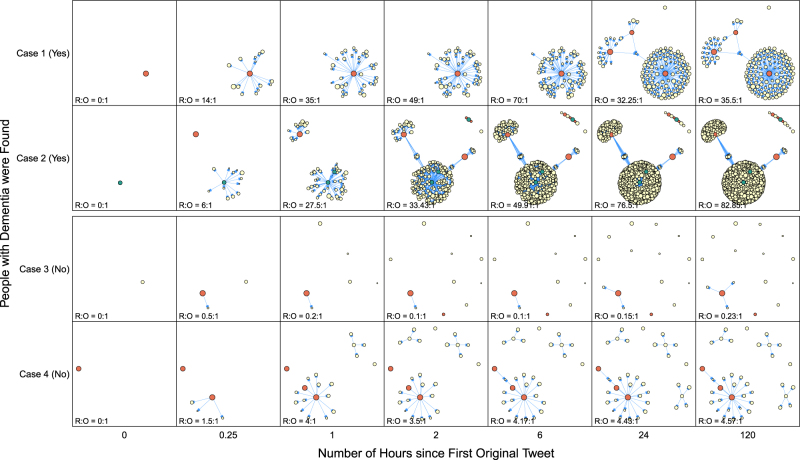
Network Graphs to show the Spread of Tweets. Four people, where two of them were found and two were not, were selected to demonstrate the spread of tweets along the follow-up time after the first original tweets. Each circle represents a tweet or retweet for the people who got lost, and its size increases with the number of followers. Tweet-writers from media organisations or police departments were presented by circles filled with red and green respectively, while the others were presented by circles filled with yellow. The R:O ratio represents the retweeted number per original tweet

In our univariate analyses, age, average number of followers, original tweets with photo or webpage link showed significant difference between the people who can be found, i.e., case group, and the people who cannot be found, i.e., control group (Table 3). Original tweets posted by police departments or media organisations, or those that mentioned 'alzheimer', 'police' or 'dementia', were marginally statistically insignificant with *p*-values of <0.20. All these variables were then fitted into a multivariate logistic regression model. The resulting stepwise model fulfilled the Hosmer–Lemeshow goodness-of-fit tests with *p*-value of 0.313. Aged 80 or above (*p* = 0.008), having original tweets posted by police departments (*p* = 0.041), and having original tweets with photo (*p* = 0.008) were the significant predictive factors that can help with finding a person who got lost. The time of people who can be found was significantly earlier when the original tweets were posted with photos (*p* = 0.043), or by police departments (*p* = 0.036) (Fig. 2). Network graphs were plotted to show four selected samples from case and control groups to demonstrate the time of message spread on Twitter (Fig. 3).

**Table 3:** Logistic regression with possible factors that help to find the people with dementia

	Odds ratio (95% CI)	*p*-value
**Univariate analyses with logistic regression**		
Age, (below 70 as reference)		
70–79	**6.76 (1.31**–**35.0)**	**0.023**
80 or above	**0.15 (0.04**–**0.60)**	**0.007**
Gender, male	2.22 (0.58–8.49)	0.243
Average no. of tweet-writers	1.09 (0.91–1.30)	0.340
Average no. of retweeters	1.01 (0.98–1.04)	0.440
Average no. of followers (in logarithmic scale)	**1.34 (1.03**–**1.74)**	**0.031**
Original tweet posted by police departments (Yes/No)	7.80 (0.92–65.8)	0.103
Original tweet posted by media organisations (Yes/No)	5.33 (1.44–19.8)	0.112
Original tweet with photo (Yes/No)	**6.75 (1.54**–**29.6)**	**0.011**
Original tweet with webpage links (Yes/No)	**5.67 (1.45**–**22.1)**	**0.012**
Original tweet with hashtags (Yes/No)	1.33 (0.39–4.55)	0.646
Original tweet that mentioned 'Alzheimer' (Yes/No)	6.26 (0.74–53.1)	0.093
Original tweet that mentioned 'dementia' (Yes/No)	0.16 (0.02–1.36)	0.093
Original tweet that mentioned 'police' (Yes/No)	3.32 (0.80–13.7)	0.098
No. of retweet / each original tweet	1.01 (0.97–1.05)	0.504
**Multivariate logistic regression model (Stepwise** ^**a**^ **)**		
Age, over 80	**0.08 (0.01**–**0.53)**	**0.008**
Original tweets posted by police departments	**25.1 (1.14**–**554)**	**0.041**
Original tweets with photo	**34.3 (2.55**–**462)**	**0.008**

Remarks:

^a^Variables with *p*-valve <0.2 from the univariate analyses were selected into the multivariate logistic regression model. The most significant predictive factors were selected by a stepwise approach where the variables were excluded one-by-one until the best fitted model is obtained with the minimum value of Akaike information criterion (AIC). All significant results (*p*-value <0.05) were shown in bold

For image processing of individual photos on Twitter, 36 and 8 photos were crawled for the lost people in case and control groups respectively. Forehead (68.2%), chin (63.6%), facial hair (63.6%) and jaw (50.0%) were the most common facial features extracted by the label detection functionality in Google’s Vision API. However, a total of six photos were misclassified. In addition, the face detection functionality of the Vision API failed to identify two photos (4.5%) to have any faces and it only recognised 22 photos (50.0%) as elderly people. We manually verified that all photos were indeed the pictures of elderly people. As the accuracy of image processing was uncertain, it was considered that the results were not reliable enough for building analyses upon them and these features on images were not fitted into the multivariate analysis model.

### Prediction score

Based on the coefficients in the resulting multivariate logistic model, the numbers of points attributed to each variable were: −2 for people who aged 80 or above, 3 for having original tweets posted by police departments, and 3 for having original tweets with a photo of the lost person. The final prediction score for a person who got lost is defined by the sum of the above scores, which would range from −2 to 6. To evaluate the performance of this score, a receiver operating characteristic (ROC) curve was plotted and the area under curve obtained was 87.5%, indicating a good prediction. Furthermore, the cutoff score of prediction was chosen to be 2. A person who scored less than 2 would be predicted not to be found and vice versa. Given this stratification, the prediction score achieved a sensitivity of 82.5% and a specificity of 83.3%.

## Discussion

To our knowledge, this is the first study that applies the methodology in epidemiological research on social media data to investigate how Twitter may be a useful tool in identifying persons with dementia who get lost. Although some features of the tweets from content analysis and image analysis did not show any statistical significance, this study opens up an important research direction, which is to use social media data on healthcare research.

Social media provides rich information about human behaviour of using the Internet. Messages in social media platforms can be transmitted much faster than the traditional ways of communication, such as population surveys, personal interviews, police updates or media reports. Nowadays, the information on social media can be easily modified to provide updated information or supplementary notes. Many existing social media research that analysed Twitter data focused on sentiment analysis or the spreading pattern of tweets, but the majority of these research did not define a quantitative end-point as the study outcome. Among those with a defined study end-point, many of them were limited to predicting election results. Consequently, we used a case–control study design to evaluate whether a lost person can be found within a month from the first tweet. If a person is found eventually, the police or media can make an announcement immediately through Twitter or other webpages. Thus, this was taken as the end-point of this study.

It is common for people with dementia to get lost while driving. Holzer et al. reported that around 30% patients with early-onset AD got lost while driving;^16^ our current study found that 10 out of 29 people (34%) got lost while driving. The time spent to find a missing person is critical, because people with a longer missing time are more likely to be harmed.^15,25^ Bowen et al. found that among the people who got lost, around half of them (43.6%) were found within 10 min and most of them were found near the place where they were last seen (61.4%).^11^ However, in this study, none of the people who got lost could be found within 10 min.

In this study, cases were defined by people with dementia who were found in 1 month. With this definition, 40 cases and 14 controls were classified. Sensitivity analysis was conducted by choosing different cut-off times to define cases and controls in order to justify our choice of cut-off. Using 1 day as the cut-off time would result in 15 cases and 39 controls. However, many factors did not show significant difference between the two groups with this cut-off. In contrast, if a later cut-off time was chosen, many more significant factors would be identified in the univariate variable selection procedure. It is believed that some features can only show significance after a certain period of time. For example, the police would only post tweets after confirming the reported lost cases are valid. Therefore, 1 month was chosen as our cut-off to ensure the case group includes all lost people who were eventually found.

The variables included in our final model are quite straightforward and explainable. Having photos in original tweets will increase the chance of finding the person because images could provide much richer information and help with portraying the person’s appearance when compared to textual descriptions. It would be much easier for police officers or the general public to search for the lost person if a photo is given. On the other hand, having tweets posted by police indicates that the police departments have recognised the case. In fact, previous research has emphasised the role of police in searching for someone who got lost due to dementia as well. A research that used missing person records in the United Kingdom found that 42.3% lost cases were found by police. Rowe’s study also indicated most lost people were found by police officers (36%) and Good Samaritans (34%) instead of the caregivers.^26^

Tweets posted by law enforcement units such as police departments are readily available. However, it is difficult to differentiate between the effects of police’s involvement in looking for the missing persons and the effect of police posting a tweet. The significance of police’s contribution might be due to their physical actions such as searching for the person on the street, or even home visits around the area, instead of their tweets. However, it should be noted that we were unable to determine whether or not the police physically searched for the missing persons. Although we were unable to conclude whether having tweets posted by the police is the primary reason of finding a lost person, police’s tweets can still be used to infer whether the police physically searched for the lost person. Therefore, one potential advantage of social media data is its availability. Besides, this may also explain the reason why the role of traditional media were not considered to be important in finding a lost person according to our final model. Although they disseminate messages on Twitter, they do not reach out to search for the lost people.

According to Twitter, hashtags are used to 'index keywords or topics'.^27^ By adding hashtags in a tweet, followers of the tweet would be able to quickly perform search queries on Twitter to view other tweets that also contain the same hashtags. As a unique feature of social media as opposed to traditional media, we would like to assess the effectiveness of using hashtags to find the people who got lost due to dementia. However, our analysis showed that using hashtags did not significantly increase the chance of finding a lost person (*p* = 0.646). One possible explanation for this is the non-standardised format and wordings in hashtags. Tweet-writers from different organisations can assign different hashtags even if they are talking about the same event, such as using full names like '#EmmaStone' vs. only using given names like '#Emma', and a lost person’s last seen street like '#MountHollywoodDrive' vs. the town last seen like '#LosAngeles'. This hinders the followers from looking for all other tweets talking about the same person.

In our study, a prediction score was developed based on the final model.^28^ It should be noted that while this method is rather uncommon in academic research related to social science and engineering, the study design is more common in medical research. Examples include using a risk score to stratify risks for colorectal advance neoplasia for Asians^29^ and mortality risk score prediction in patients with unstable angina or c.^30^ As opposed to applying the sophisticated full model for prediction, the primary reason of using a prediction score is to make it intuitive and simple to use. Similar to the risk scores mentioned in the examples above, our prediction score can be applied by simply answering a few yes–no questions followed by doing some simple additions.

We attempted to apply image analysis in this study. Although it was reported that 93.9% of the labels detected by Google Cloud’s Vision API were relevant,^31^ the labels detected in this study were found to be not satisfactory enough. Also, the results of image analysis were not unreliable enough for us to incorporate them into the multivariate regression model. The unsatisfactory performance could be due to the quality of photos. The photos were being crawled from Twitter, police sites or media sites tend to be rather small in size because running image compression is a usual practice in web applications to spare storage space. Twenty-eight (63.6%) of the photos in this study were smaller than 640 times 480 pixels in size, which would be more difficult for algorithms to extract useful features from them. In addition, eleven (25%) photos were considered to be blurry. While human can still easily recognise the facial features of the person in the photos, algorithms would find it more difficult to do so. In fact, image analysis web services, such as Google’s Vision API used in this study, were seldom explored and applied in academic research despite being a potentially very powerful tool. Little has been done to evaluate the accuracy of these image analysis services. Future studies may work on comparing their accuracies and after that, the one that can best adapt to smaller images could be applied to this study.

There are some limitations in our study. First, the findings of this study may not be applicable to other social media platforms, as the effectiveness of tweeting might be exaggerated by the 'Silver Alert' programmes in the United States. 'Silver Alert' programmes are set up to rescue elderlies when they get lost. Once an elder is reported missing, the programme would immediately notify the neighbourhood and provide information to law enforcement agencies and third parties such as the department of transportation and media outlets. These alert programmes have been proven to be effective.^17^ A person that was successfully found might be attributed more to the programme instead of messages on Twitter. Second, tweets were only extracted from April and May in 2017. If the work were to be extended to a yearly basis, a huge database of tweets can be created. Although natural language processing was used to speed up the process of identifying lost cases, the manpower required to manually inspect the outcome of searching efforts will increase drastically. This workload is deemed to be unaffordable for us. We are however confident that this pilot study can encourage more researchers to conduct similar social media research with well-defined study end-points. Finally, it should be noted that our methods of collecting tweets cannot perfectly extract all tweets in interest due to limitations of the API. Query search in Twitter’s REST API was used in this study, which was described to be focusing more on relevance but not completeness.^32^ In other words, the outputs from the API is not guaranteed to be complete.

Throughout this study, it was also noticed that many users were still tweeting or retweeting the information about the lost persons even after they were found, which is a waste of Internet resources. Our data showed that after persons who got lost were found, 1176 relevant tweets were still posted, which were composed by 1142 (97.1%) retweets and 34 (2.9%) original tweets. Retweeting can be as easy as clicking a button, but people seldom assure the correctness of the messages before posting. This idea can also be shown in various other studies. A study by Gupta et al. on Twitter message spread showed at during a hurricane, 10,350 tweets (64.2%) with fake images and 5767 tweets (35.8%) with real images were posted.^33^ Among those tweets with fake images, a majority of them (86%) were retweets. Another study by Starbird et al. looked into three cases to illustrate that rumours and misinformation could be widely spread on Twitter.^34^ To avoid the waste of Internet resources, we would suggest social media users to check for the latest updates before tweeting or retweeting.

Our results are of interest to the caregivers who have family members with dementia. Chenoweth and Spencer revealed that the largest problem for family caregivers was to provide constant physical care and supervision to dementia patients; being a caregiver also adversely affected their mental health.^35^ By providing advices to caregivers using our results, they will know better ways of using Twitter to search for their lost relatives with dementia and this would hopefully relieve the stress they are facing. In the long run, we believe the involvement of Twitter can help these caregivers. To the best of our knowledge, Twitter currently do not take any specific actions in order to spread the message of someone being lost. Therefore, when a police department tweets about a lost person, only their followers would be able to see those tweets. If Twitter could push those tweets posted by authorities to people who live around the area, this would certainly help with spreading the message and hence increase the chance of finding the lost people.

In conclusion, this study showed that social media, such as Twitter, can help with searching a person who got lost due to dementia. Young age, having tweets with photos, and having tweets posted by police departments were all found to significantly increase the chance of finding the missing persons. Although results from content analysis on tweets and image analysis on photos did not show significant association with the chance of finding the lost person, this study opens up an important research direction in the era of big data. The future use of advanced technology and computing algorithms can strengthen the application of social media data, and even extend the feasibility of interdisciplinary research.

## Methods

### Study design

This is a retrospective cas–control study design to investigate the associating features of tweets on Twitter to find someone who got lost due to dementia. An individual who can be found in a month were defined as the case, and those who cannot be found were defined as the control. Features of the tweets were compared between case and control subjects.

### Data collection

The messages on Twitter, i.e., tweets, were collected through API.^36^ All tweets that were related to people who got lost due to dementia or Alzheimer were downloaded at Greenwich mean time 8 am every day from April 1 to May 30, 2017 with an R script through Twitter REST APIs using *rtweet* package in R version 3.3.3.^37,38^ Daily collection of tweets was to ensure the data traffic volume was within the API rate limits.^39^ Some samples of tweets are listed in Supplementary Table 1. Any person who got lost in April was identified. All tweets were sorted by time^40^ and followed up for a month after the first original tweet.

### Database construction

Users who created original tweets were identified as tweet-writers, while other users were classified as retweeters if they reposted the tweets, or followers if they only read the posts. The flow of message spread can be traced by the creation time of individual posts and user ID of the tweet-writer. Original tweets were manually reviewed by the researchers (N.B.C., L.Z.) to inspect whether or not the tweets were posted with photos.

Twitter users were classified into police departments, media organisations, or neither. If the screen name or account description contains any keywords related to police, sheriff, or public safety, the researchers would manually study whether the source of tweets were from a police department. Similarly, when the account information contains any keywords related to media, radio, news, TV, FM, daily or weekly posts, the researcher would check and see whether it was from a media organisation. In addition, the background of tweet-writers who have over a thousand followers were checked.

### Identification for case and control

In the tweet database, natural language processing was conducted on the text of the original tweets with an R package *openNLP*.^41^ As a result, the lost people’s details, including name, age, and lost location, were captured. The name of individuals mentioned in the original tweets with any two of the details successfully captured were further reviewed by the researcher for confirmation.

Once the list of people who got lost was gathered, their other personal characteristics, including gender, country of origin, and activity before the lost, were manually extracted by inspecting the external webpages linked from the original tweets. The name and location of each lost person were then searched throughout all original tweets to obtain a full list of original tweets for each lost person. After that, the corresponding retweets and user information of these original tweets were extracted. As a result, all tweets of the people who got lost and posted on Twitter were identified.

To summarise, the database of tweets included the following information:

i. Types of users: Tweet-writers, retweeters and followers;
ii. Sources of Twitter accounts: police departments, media organisations, or the general public;
iii. Features of tweets: original tweets, retweets, tweets with webpage links, photos, or hashtags, textual contents of the tweets;
iv. Characteristics of the person who got lost: age, gender, country of origin, and activities before the lost, such as walking on the street or driving.

### Inclusion and exclusion criteria

All tweets containing keywords 'missing/lost' and 'dementia/Alzheimer' in April 2017 were included. The original tweets were excluded if: (i) tweets were written in languages other than English; (ii) tweets were posted in April, but the person got lost in March; (iii) the exact name of the lost person cannot be identified; (iv) the person was found before the first original tweet was posted.

### Outcome

Any person who can be found in a month after the posting time of the first original tweet on Twitter was defined as the outcome of this study. All external webpage links from the original tweets were checked to confirm these lost cases. Furthermore, the names of individuals were searched on Google for updates. For the cases who cannot be found in a month, their individual updates were further checked until 90 days after the first original tweets to confirm they were not found, but no one was found beyond a month eventually.

### Variables

The average numbers of tweet-writers, retweeters, and followers of the people who got lost were compared between case and control groups. The number of followers across Twitter users differ significantly, so the number of followers was transformed to a logarithmic scale. Twitter users from police departments or media organisations were fitted as binary variables in the prediction model.

For Twitter posts, only tweets within a certain period of time was counted. For the case group, all tweets were counted until the lost person was found. For the control group, as they cannot be found, the average found time of the people in case group was taken as the cutoff time for the control group. The number of tweets were averaged by the number of people in case and control groups respectively. Furthermore, original tweets with photos, webpage links, or hashtags were also labelled for model fitting.

Age and gender of the person who got lost were defined as the personal predictors. With a limited sample size, age was categorised into aged below 70, age between 70 and 79, or aged 80 or above. The tweets posted in different countries were considered. However, as almost half of the cases had no information on their activities before getting lost, these factors were only used in descriptive statistics but not in predictive models.

Content analysis was performed to quantify the word patterns across the original tweets. All text embedded in the original tweets were first cleansed by removing special characters and stop words such as ‘at’, ‘a’ and ‘the’. After that, individual words were stemmed and a term-document matrix was then generated by counting the number of occurrences of each word. They served as features of tweets. On the other hand, to describe the speed of message spread on Twitter, the timestamp of each retweet was used. The number of retweets from the first original tweet was calculated at different time points. A ratio was used to quantify the number of retweets per original tweet, i.e., R:O ratio. The patterns of message spread among Twitter users across time were plotted with network graphs.

If the photo of the people who got lost was available in the tweets, they were downloaded for image analysis. Image analysis was done by using Google Cloud’s Vision API.^42^ Equipped with the API, features of the photos were extracted and expressed as specific labels, such as ‘human’, ‘trees’, or ‘buildings’. All labels extracted from the photos were reported with a confidence score, which ranges from 0 to 1. A higher confidence score represents a higher likelihood of existence in the photo. Only labels with a confidence score greater than 0.5 were selected as the features in our multivariate analysis.

### Data analyses

Descriptive statistics were used to summarise the features of the lost people, Twitter users, and tweets between the case and control groups. All variables were compared by *Χ*^2^ test or independent sample *t*-test whichever appropriate. A two-tailed *p*-value of <0.05 was considered to be statistically significant. For content analysis, frequently-used words in either groups were shortlisted and univariate logistic regression were performed to see if their relationships with the outcome were significant. Univariate analyses were carried out to identify the potentially important variables. Variables with *p*-value <0.2 in the univariate analyses were included in multivariate logistic regression model. The most significant variables were then selected by a stepwise approach where the variables were excluded one-by-one until the best fitted model is obtained. The multivariate models was compared by selecting the minimum value of AIC, and the Hosmer–Lemeshow goodness-of-fit test was applied to evaluate whether or not it is a good fit. The goodness-of-fit is rejected when the *p*-value <0.05. All variables in multivariate analyses were incorporated into a predictive score with reference to the estimated coefficients of the best fitted model. The coefficients were rounded to the nearest integer, and the validity of the score was assessed by a ROC curve. Area under the ROC curve was used to illustrate the predictive ability of the model for the people who can be found. Kaplan–Meier (KM) curves were plotted to compare the time of people who can be found with the significant binary variables in the multiple logistic regression model. Log-rank tests were performed to distinguish the difference between the two KM curves with *p*-value <0.05 as statistically significant. For image analysis, the number of cases that contained each tag were counted. *Χ*^2^ tests were then performed to determine the potential effect of each tag on the searching outcome. All statistical analyses, data visualisation and content analysis were conducted by R version 3.3.3,^38,43–47^ with the exception of Python being used in calling Google’s API for image analysis. An excerpt of the programming codes used were listed in Supplementary Table 2 for reference.

### Data availability

All relevant data that support the findings of this study are available in figshare with the identifier 10.6084/m9.figshare.5788125.^48^

## Electronic supplementary material

Supplementary Table 1(DOCX 26 kb)

Supplementary Table 2(DOCX 36 kb)

## Electronic supplementary material

**Supplementary information** accompanies the paper on the *npj Digital Medicine* website (10.1038/s41746-018-0017-5).

## References

[1] Hunt LA, Brown AE, Gilman IP (2010). Drivers with dementia and outcomes of becoming lost while driving. Am. J. Occup. Ther..

[2] Prince M (2013). The global prevalence of dementia: a systematic review and metaanalysis. Alzheimers Dement..

[3] Rowe MA, Bennett V (2003). A look at deaths occurring in persons with dementia lost in the community. Am. J. Alzheimers Dis. Other Dement..

[4] The World Health Organization. *10 Facts on Dementia* http://www.who.int/features/factfiles/dementia/en/ (2017).

[5] Winblad B (2016). Defeating Alzheimer’s disease and other dementias: a priority for European science and society. Lancet Neurol..

[6] Chan KY (2013). Epidemiology of Alzheimer’s disease and other forms of dementia in China, 1990–2010: a systematic review and analysis. Lancet.

[7] Roth ME (1993). Advances in Alzheimer’s disease: a review for the family physician. J. Fam. Pract..

[8] Brookmeyer R, Johnson E, Ziegler-Graham K, Arrighi HM (2007). Forecasting the global burden of Alzheimer’s disease. Alzheimers Dement..

[9] Agüero-Torres H (1998). Dementia is the major cause of functional dependence in the elderly: 3-year follow-up data from a population-based study. Am. J. Public Health.

[10] Lewis H (2002). Dementia in New Zealand: Improving Quality in Residential Care. A report to the Disability Issues Directorate, Ministry of Health.

[11] Bowen ME, McKenzie B, Steis M, Rowe M (2011). Prevalence of and antecedents to dementia-related missing incidents in the community. Dement. Geriatr. Cogn. Disord..

[12] McShane R (1998). Getting lost in dementia: a longitudinal study of a behavioral symptom. Int. Psychogeriatr..

[13] Hwang JP, Yang CH, Tsai SJ, Liu KM (1997). Behavioural disturbances in psychiatric inpatients with dementia of the Alzheimer’s type in Taiwan. Int J. Geriatr. Psychiatry.

[14] Kwok TC, Yuen KS, Ho FK, Chan WM (2010). Getting lost in the community: a phone survey on the community‐dwelling demented people in Hong Kong. Int J. Geriatr. Psychiatry.

[15] Koester RJ, Stooksbury DE (1998). The lost Alzheimer’s and related disorders search subject: new research and perspectives. Response.

[16] Holzer C, Warshaw G (2000). Clues to early Alzheimer dementia in the outpatient setting. Arch. Fam. Med..

[17] Rowe MA, Greenblum CA, Boltz M, Galvin JE (2012). Missing drivers with dementia: antecedents and recovery. J. Am. Geriatr. Soc..

[18] Kirkman AM (2006). Dementia in the news: the media coverage of Alzheimer’s disease. Australas. J. Ageing.

[19] Rowe MA (2011). Persons with dementia missing in the community: is it wandering or something unique?. BMC Geriatr..

[20] eMarketer. *Number of Social Media Users Worldwide from 2010 to 2021 (in billions)* https://www.statista.com/statistics/278414/number-of-worldwide-social-network-users/ (2017).

[21] Obar JA, Wildman SS (2015). Social media definition and the governance challenge: An introduction to the special issue. Telecommun. Policy.

[22] Twitter. *About Twitter* https://about.twitter.com/company (2017).

[23] Twitter Developer Documentation. *API Overview* https://dev.twitter.com/overview/api (2017).

[24] Facebook for developers. *The Graph API*. https://developers.facebook.com/docs/graph-api (2017).

[25] Bantry White E, Montgomery P (2016). Supporting people with dementia to walkabout safely outdoors: development of a structured model of assessment. Health Soc. Care Community.

[26] Rowe MA (2003). People with dementia who become lost: preventing injuries and death. AJN Am. J. Nurs..

[27] Twitter. *Using hashtags on Twitter* https://support.twitter.com/articles/49309 (2017).

[28] Tsoi, K. K. F. et al. Social media as a tool to look for people with dementia who become lost: factors that matter. In *Proc. 51th Hawaii International Conference on System Sciences* 3355–3364 (IEEE, Hawaii, 2018).

[29] Yeoh KG (2011). The Asia-Pacific colorectal screening score: a validated tool that stratifies risk for colorectal advanced neoplasia in asymptomatic Asian subjects. Gut.

[30] Morrow DA (2000). TIMI risk score for ST-elevation myocardial infarction: a convenient, bedside, clinical score for risk assessment at presentation. Circulation.

[31] Casalboni A. *Google Vision vs. Amazon Rekognition: A Vendor-neutral Comparison* https://cloudacademy.com/blog/google-vision-vs-amazon-rekognition/ (2017).

[32] Twitter. *Twitter Developer Documentation-The Search API* https://dev.twitter.com/rest/public/search (2017).

[33] Gupta, A., Lamba, H., Kumaraguru, P., & Joshi, A. Faking sandy: characterizing and identifying fake images on twitter during hurricane sandy. In *Proc. 22nd international conference on World Wide Web* 729–736 (ACM, Rio de Janeiro, Brazil, 2013).

[34] Starbird, K., Maddock, J., Orand, M., Achterman, P., & Mason, R. M. Rumors, false flags, and digital vigilantes: Misinformation on twitter after the 2013 Boston marathon bombing. In *iConference 2014 Proceedings* (iSchools, Berlin, Germany, 2014).

[35] Chenoweth B, Spencer B (1986). Dementia: The experience of family caregivers. Gerontologist.

[36] Twitter Developer Documentation. *REST APIs* https://dev.twitter.com/rest/public (2017).

[37] Kearney, M. W. *rtweet: Collecting Twitter Data, R package version 0.4.0* https://cran.r-project.org/package=rtweet (2017).

[38] R Foundation for Statistical Computing. *R: A language and environment for statistical computing* https://www.R-project.org/ (2017).

[39] Twitter Developer Documentation. *API Rate Limits* https://dev.twitter.com/rest/public/rate-limiting (2017).

[40] Twitter Developer Documentation. *GET Search/Tweets* https://dev.twitter.com/rest/reference/get/search/tweets (2017).

[41] Hornik, K. *openNLP: Apache OpenNLP Tools Interface, R Package Version 0.2-6* https://CRAN.R-project.org/package=openNLP (2017).

[42] Google. *Vision API* https://cloud.google.com/vision/ (2017).

[43] Feinerer, I. & Hornik, K. *tm: Text Mining Package, R Package Version 0.7-1* https://CRAN.R-project.org/package=tm (2017).

[44] Fellows I. *wordcloud: Word Clouds, R Package Version 2.5* https://cran.r-project.org/web/packages/wordcloud/ (2017).

[45] Csardi G, Nepusz T (2006). The igraph software package for complex network research. Inter. Complex Syst..

[46] Lemon J (2006). Plotrix: a package in the red light district of R. R. News.

[47] Wickham, H., Chang, W. *ggplot2: Create Elegant Data Visualisations Using the Grammar of Graphics, R Package Version 2.2.1* https://cran.r-project.org/web/packages/ggplot2/ (2017).

[48] Tsoi, K. K. F. et al. A twitter dataset on tweets about people who got lost due to dementia. *figshare*. 10.6084/m9.figshare.5788125.v1 (2018).

