# Supplementary material for: How can we better use Twitter to find a person who got lost due to dementia?
Source: NPJ Digit Med. 2018 Apr 18;1:14. doi: 10.1038/s41746-018-0017-5 (PMC6550184; doi:10.1038/s41746-018-0017-5)
Supplement: Supplementary file 1 — Supplementary Table 1(DOCX 26 kb) [file 41746_2018_17_MOESM1_ESM.docx]

**Supplementary Table 1. Examples of Tweets that Looks for a Person who Got Lost due to Dementia**

| **Content Type** | **Source** | **Example** |
| --- | --- | --- |
| Original Tweet | Police | MISSING ADULT ALERT: [name], 83, w/ early onset dementia. Last seen at 4 p.m. in [place] [URL] |
|  | Media | MISSING PERSON: 65 yr old woman missing since 4pm wearing black dress last seen on [place]. Suffers from dementia.… [URL] |
|  | General Users | [place] Police looking for missing man with dementia [URL] via @10tv #[place] #MissingPerson |
| Further Announcement | Police | UPDATE: Missing Person At Risk FOUND. 75-year-old [name] was found safe in [place]. |
|  | Media | Missing [place] man with Alzheimer's located, [place] police say [URL] |
|  | Tweet-writers | UPDATE: Golden Alert canceled for missing [place] man with Alzheimer's. [name] was found safe tonight |

Remarks:

The names of the lost people, places where the people got lost and external webpage links are censored here for privacy. They are replaced with type of the information enclosed with a pair of square brackets.
