# Supplementary material for: How can we better use Twitter to find a person who got lost due to dementia?
Source: NPJ Digit Med. 2018 Apr 18;1:14. doi: 10.1038/s41746-018-0017-5 (PMC6550184; doi:10.1038/s41746-018-0017-5)
Supplement: Supplementary file 2 — Supplementary Table 2(DOCX 36 kb) [file 41746_2018_17_MOESM2_ESM.docx]

**Supplementary Table 2. List of Programming Packages Used and Code Excerpt**

| **Name** | **Short Description** | **Code** **Excerpt** |
| --- | --- | --- |
| **R Packages** |  |  |
| twitteR | For extracting tweets and user info from Twitter | library(*rtweet*)  # define function to crawl tweets  **gtweet**<-function(*keyword*){  tryCatch({  *res* **<-** search_tweets(*keyword*, *n* **=** *10000000*, *since* **=**Sys.Date()**-***1*,  *retryonratelimit*=*TRUE*, *type* **=** "recent",*include_rts* **=** *T*)  }, *error* **=** **function**(*e*){  print(*e*)  Sys.sleep(*60*******15*)  }, *warning* = **function**(*w*){  print(*w*)  Sys.sleep(*60*******15*)  })  **return**(*res*)  } |
| dplyr  magrittr  stringr  lubridate | For general data manipulation such as filtering, grouping, matching strings, and doing operations on dates and time | library(*dplyr*); library(*magrittr*)  # filter out non-english tweets  *tweets* **<-** *tweets* %**>**% filter(*lang***==**"en")  # count the number of original tweets and retweets crawled  *tweets* %**>**% group_by(*is_retweet*) %**>**% summarize(*count*=n()) %**>**% *print* |
| NLP  openNLP  tm | For natural language processing | # require all packages  lapply(c('NLP', 'openNLP', 'tm', 'stringr'), *require*, *character.only* **=** *TRUE*)  # Natural language processing for original tweets using openNLP  *word_ann* **<-** Maxent_Word_Token_Annotator()  *sent_ann* **<-** Maxent_Sent_Token_Annotator()  *person_ann* **<-** Maxent_Entity_Annotator(*kind* **=** "person")  *location_ann* **<-** Maxent_Entity_Annotator(*kind* **=** "location")  *pipeline* **<-** list(*sent_ann*, *word_ann*, *person_ann*, *location_ann*)  # Extract entities from an AnnotatedPlainTextDocument  **entities** **<-** **function**(*doc*, *kind*) {  *s* **<-** *doc***$***content*  *a* **<-** annotations(*doc*)[[*1*]]  **if**(hasArg(*kind*)) {  *k* **<-** sapply(*a***$***features*, ‘[[‘, "kind")  *s*[*a*[*k* **==** *kind*]]  } **else** {  *s*[*a*[*a***$***type* **==** "entity"]]  }  }  # only use the original tweets for case identification  *tweets.ot* **<-** *tweets* %**>**% filter(*is_retweet***==***F*)  # prepare data frame to storing results  *nlpresult* **<-** data.frame(*status_id*=*NA*, *age*=*NA*, *name*=*NA*, *place*=*NA*)  # for the text in each original tweet, use openNLP to try to extract information from it  **for**(*i* **in** *1***:**nrow(*tweets.ot*)){  *bio* **<-** *tweets.ot*[*i*, 'text']  *bio_annotations* **<-** *NLP***::**annotate(*bio*, *pipeline*)  *bio_doc* **<-** AnnotatedPlainTextDocument(*bio*, *bio_annotations*)  *temp_person* **<-** entities(*bio_doc*, *kind* **=** "person")  *temp_loc* **<-** entities(*bio_doc*, *kind* **=** "location")  *temp_age* **<-** str_match(*bio*, "([0-9]+)( \|-)*(year\|yr)") # Regex is being used for age  *nlpresult*[*i*, 'status_id'] **<-** *rawtweets.ot*[*i*, 'status_id']  *nlpresult*[*i*, 'name'] **<-** ifelse(length(*temp_person*)**>***0*, as.character(*temp_person*), *NA*)  *nlpresult*[*i*, 'place'] **<-** ifelse(length(*temp_loc*)**>***0*, as.character(*temp_loc*), *NA*)  *nlpresult*[*i*, 'age'] **<-** *temp_age*[*2*]  }  # only tweets w/ at least 2 pieces of information (among age, name and place) are inspected manually  *nlpresult.suff* **<-** *nlpresult*[as.vector(apply(*nlpresult*[,c('name','place','age')], *1*, **function**(*row*){**return**(sum(*1****!**is.na(*row*)))})) **>=** *2*,]  *nlpresult.suff* %**>**% left_join(*rawtweets*, *by*='status_id') %**>**% arrange(*name*, *place*, *age*, *created_at*) %**>**% *View* |
| ggplot2 | For plotting general graphs |  |
| wordcloud | For plotting wordclouds | library(*wordcloud*)  par(*mfrow*=c(*1*,*2*))  comparison.cloud(*term.matrix.found*, *max.words*=*45*, *random.order*=*FALSE*)  comparison.cloud(*term.matrix.notfound*, *max.words*=*45*, *random.order*=*FALSE*) |
| igraph | For plotting network graphs | library(*igraph*)  # initialize network graph with original tweets that have retweets  *net* **<-** graph.data.frame(*node_hv.rt*,*attr_rt*,*directed*=*T*)  # add original tweets that have NO retweets  *net* **<-** add_vertices(*net*, *nv* **=** nrow(*node_no.rt*), *attr* **=** list(*name* **=** *node_no.rt***$***screen_name*))  plot(*net*,  # colour of node depends on user type: police/media/other  *vertex.color* **=** c("#E76F51", "#2A9D8F", "#ffffcc")[factor(V(*net*)**$***occupation*)],  *vertex.label*=*NA*,  # size of node depends on user's number of followers  *vertex.size* **=** log(V(*net*)**$***followers_count***+***1*),  *layout*=layout.fruchterman.reingold(*net*,*grid*="nogrid", *niter*=*20000*))  ) |
| **Python Packages** | | |
| google.cloud | For assessing Google Cloud’s client library | **import** io  **import** os  **import** matplotlib.pyplot **as** plt  **import** numpy **as** np  **import** pandas **as** pd  **from** PIL **import** Image  # Imports the Google Cloud client library  **from** google.cloud **import** vision  **from** google.cloud.vision **import** types  # Instantiates a client  client **=** vision.ImageAnnotatorClient()  # Prepare df for storing result  resultdf **=** pd.DataFrame(*columns***=**['name', 'photocode','label', 'score'])  # Loop through files in directory  **for** **file** **in** os.listdir(os.path.join(os.path.dirname(**__file__**),'Images')):  # The name of the image file to annotate  file_name **=** os.path.join(os.path.dirname(**__file__**),'Images', **file**)  **print**(**file**)    # check if it is a photo  **if** (file_name.split('.')[**-***1*].lower() **in** ['jpg', 'jpeg', 'png']):    # Loads the image into memory  **with** Image.open(file_name, 'r') **as** pil_im:  plt.axis('off')  plt.imshow(np.asarray(pil_im))  plt.show()    **with** io.open(file_name, 'rb') **as** image_file:  content **=** image_file.read()    image **=** types.Image(*content***=**content)    # Performs label detection on the image file  response **=** client.annotate_image({  'image': {'content': image.content},  'features': [{'type': vision.enums.Feature.Type.LABEL_DETECTION, "max_results":*100*}],  })  result **=** response.label_annotations    **for** label **in** result:  **print**("*%20s* *%5f*" **%** (label.description, label.score))  resultdf **=** resultdf.append(pd.DataFrame([[**file**.split('_')[*0*], **file**.split('_')[*1*][*0*],  label.description, label.score]],  *columns***=**['name', 'photocode','label',  'score']),  *ignore_index***=***True*)  **print**('------------------------------')  # Export as CSV  resultdf.to_csv('label_result.csv', *index* **=** *False*) |
